# Electronic cigarette aerosols suppress cellular antioxidant defenses and induce significant oxidative DNA damage

**DOI:** 10.1371/journal.pone.0177780

**Published:** 2017-05-18

**Authors:** Vengatesh Ganapathy, Jimmy Manyanga, Lacy Brame, Dehra McGuire, Balaji Sadhasivam, Evan Floyd, David A. Rubenstein, Ilangovan Ramachandran, Theodore Wagener, Lurdes Queimado

**Affiliations:** 1 Department of Otorhinolaryngology, The University of Oklahoma Health Sciences Center, Oklahoma City, Oklahoma, United States of America; 2 Department of Cell Biology, The University of Oklahoma Health Sciences Center, Oklahoma City, Oklahoma, United States of America; 3 Department of Occupational and Environmental Health, The University of Oklahoma Health Sciences Center, Oklahoma City, Oklahoma, United States of America; 4 Department of Biomedical Engineering, Stony Brook University, New York, New York, United States of America; 5 Department of Endocrinology, Dr. ALM Post Graduate Institute of Basic Medical Sciences, University of Madras, Taramani Campus, Chennai, Tamil Nadu, India; 6 Department of Pediatrics, The University of Oklahoma Health Sciences Center, Oklahoma City, Oklahoma, United States of America; 7 Oklahoma Tobacco Research Center, The University of Oklahoma Health Sciences Center, Oklahoma City, Oklahoma, United States of America; 8 The Peggy and Charles Stephenson Cancer Center, The University of Oklahoma Health Sciences Center, Oklahoma City, Oklahoma, United States of America; H. Lee Moffitt Cancer Center & Research Institute, UNITED STATES

## Abstract

**Background:**

Electronic cigarette (EC) aerosols contain unique compounds in addition to toxicants and carcinogens traditionally found in tobacco smoke. Studies are warranted to understand the public health risks of ECs.

**Objective:**

The aim of this study was to determine the genotoxicity and the mechanisms induced by EC aerosol extracts on human oral and lung epithelial cells.

**Methods:**

Cells were exposed to EC aerosol or mainstream smoke extracts and DNA damage was measured using the primer anchored DNA damage detection assay (q-PADDA) and 8-oxo-dG ELISA assay. Cell viability, reactive oxygen species (ROS) and total antioxidant capacity (TAC) were measured using standard methods. mRNA and protein expression were evaluated by RT-PCR and western blot, respectively.

**Results:**

EC aerosol extracts induced DNA damage in a dose-dependent manner, but independently of nicotine concentration. Overall, EC aerosol extracts induced significantly less DNA damage than mainstream smoke extracts, as measured by q-PADDA. However, the levels of oxidative DNA damage, as indicated by the presence of 8-oxo-dG, a highly mutagenic DNA lesion, were similar or slightly higher after exposure to EC aerosol compared to mainstream smoke extracts. Mechanistically, while exposure to EC extracts significantly increased ROS, it decreased TAC as well as the expression of 8-oxoguanine DNA glycosylase (OGG1), an enzyme essential for the removal of oxidative DNA damage.

**Conclusions:**

Exposure to EC aerosol extracts suppressed the cellular antioxidant defenses and led to significant DNA damage. These findings emphasize the urgent need to investigate the potential long-term cancer risk of exposure to EC aerosol for vapers and the general public.

## Introduction

Electronic cigarettes (ECs) are battery-powered devices that heat up a solution of chemicals (e-liquid) with or without nicotine and turn it into an inhalable aerosol. Whether ECs are a safer alternative to combustible tobacco products and/or assist patients with smoking cessation are still major controversies [1–6]. Nonetheless, the use of ECs has increased sharply since 2003 [7–9]. In a 2015 survey, about 10% of U.S. adults reported to use ECs [10]. Disturbingly, the use of ECs among middle and high school students has had a 4-fold increase between 2013 and 2014 reaching 3.9% and 13.4%, respectively [11]. The retail EC industry is projected to reach $50 billion USD by 2025 [12]. The U.S. Food and Drug Administration (FDA) has called for additional scientific research to inform the development of effective EC regulations [13] and has extended its regulatory authority to cover all tobacco products, including ECs [14]. The high prevalence of EC use, the polarized views on the subject, and the limited toxicology data available on EC aerosols, all stress the urgent need for rigorous evaluation of the health effects of EC aerosols to ensure public safety and support evidence-based public health policies and regulations.

The potential long-term human health effects of EC aerosols are unknown. EC aerosol constituents comprise some toxicants and carcinogens present in cigarette smoke, in addition to other unique, and potentially harmful compounds such as silicate beads, tin, and flavorants, most of which are not yet well characterized [15–18]. Chemicals identified in EC aerosols include the most potent carcinogenic tobacco-specific nitrosamines [nicotine metabolites: 4-(methylnitrosamino)-1-(3-pyridyl)-1-butanone (NNK) and N'-nitrosonornicotine (NNN)], aldehydes, volatile organic compounds, phenolic compounds, polycyclic aromatic hydrocarbons, tobacco alkaloids, heavy metals, flavors, and nicotine [15–23].

Initial studies, focused mainly on first generation and/or low power devices, reported that the levels of potentially toxic compounds in EC aerosol (e.g. formaldehyde, acetaldehyde, acrolein, and toluene) are significantly lower (9- to 450-fold lower) than those in cigarette smoke [20, 24], and in many cases (e.g., NNN and NNK) comparable with the trace amounts present in nicotine replacement products [25–27]. However, recent studies, have shown that specific toxicants and carcinogens present in EC aerosols can reach levels equal (e.g., acetaldehyde and chromium) to or exceeding (e.g., formaldehyde, and nickel) to those found in cigarette smoke, particularly as the power of the device increases [15, 22]. Formaldehyde is a human carcinogen causally associated with many cancers, including oral and lung cancer [28]. Acetaldehyde is classified as possibly carcinogenic to humans [28]. Lead, nickel, and chromium are in the FDA's “harmful and potentially harmful chemicals” list [29] and tin is a potential lung carcinogen [30, 31]. EC aerosols also contain high levels of free radicals [32–34] and have been shown to induce oxidative stress and inflammation in mouse models [32, 33]. These data suggest that EC aerosols expose users and bystanders to toxic and carcinogenic substances, which have the potential to induce DNA damage and increase cancer risk.

In this work, we investigated the effects of short-term and long-term exposure to EC aerosol extracts on the levels of DNA damage in human oral and lung epithelial cells. Given the promoted potential role of EC as a harm reduction strategy for smoking cessation, we also performed side-by-side toxicology and mechanistic studies to characterize the genotoxicity associated with exposure to EC aerosol and tobacco smoke extracts. In this study, several systems important for understanding the mechanisms of genotoxicity (e.g., total cellular antioxidant capacity, cellular reactive oxygen species, and distinct DNA repair mechanisms) were investigated taking into consideration the potential use of EC as a smoking cessation aid. Our studies identified significant differences and similarities in the mechanisms and the levels of DNA damage induced by EC aerosol and mainstream tobacco smoke. Of high public health significance, our study is the first to suggest that even very low levels of exposure to EC aerosol can lead to significant DNA damage and potentially increase cancer risk. Our study also emphasizes the urgent need to further investigate the health consequences of exposure to EC aerosols and highlights the extreme importance of regulating ECs for the purpose of protecting the public.

## Material and methods

### E-cigarette aerosol and tobacco smoke extracts

Tobacco smoke extracts were prepared from Marlboro 100s (16 mg tar and 1.2 mg nicotine, Philip Morris) cigarettes as previously described [35]. According to 2015 sales data, Marlboro is the most popular cigarette brand in the United States, with sales greater than the next eight leading competitors combined [36]. Smoking conditions were two 50 mL puffs per minute, until the cigarette burned to 3mm short of the filter. This puff regimen (volume and interval) mimics the reported human puffing profiles for cigarettes with more than 14 mg tar [37] and is similar to the Health Canada Intensive (HCI) smoking standard conditions (two 55±5 ml puffs per minute). A similar puffing regimen of two 55mL puffs per minute has been recommended for EC aerosol studies [38]. Mainstream (MS) smoke is the material drawn from the mouth end of a cigarette during puffing and inhaled by smokers. A modification of the smoke extraction apparatus was used to produce ECs extracts as previously described [39]. The changes in mass observed for ECs were consistent with the amount of EC liquid consumed by experienced EC users [40]. Five distinct EC extracts were prepared from two distinct device types: NJoy [OneJoy, Traditional Flavor, propylene glycol/vegetable glycerin (PG/VG) 50:50, undisclosed power], and eGo-T (OKC Vapes, Desert Sands Flavor, PG/VG 50:50, 6 W) and based on the commercially available nicotine concentrations for each e-liquid: N12 (NJoy 12 mg/ml nicotine), N18 (NJoy 18 mg/ml nicotine), E0 (eGo 0 mg/ml nicotine), E12 (eGo 12 mg/ml nicotine), and E18 (eGo 18 mg/ml nicotine). An e-liquid without nicotine was not commercially available for the NJoy device used in this study. Identical extraction apparatus were used for each EC device (NJoy and eGo-T). The extraction apparatus used for each device were extensively cleaned between extractions and the lines carrying EC aerosol were replaced. To assure extract stability, extracts were aliquoted and frozen at -80°C immediately after preparation. A new aliquot was thawed just before cells were to be exposed. For our two weeks exposure experiments, aliquots were maintained at -80°C, and a new aliquot was thawed every other day, just before media preparation and exchange.

Nicotine concentration was determined by Gas Chromatography Mass Spectroscopy (GCMS) analysis essentially as previously described [41], except for the use of a smaller (500 μl) sample volume (full details in S1 File). Analysis was conducted using an Agilent 6890 GC with 5973 quadrapole Mass Selective Detector. The method detection limit (MDL) of 0.076 μg/ml was estimated from 11 replicate analyses of nicotine in HEPES at 0.39 μg/ml using the EPA approach. Lower limit of quantification was set a 3MDL = 0.227 μg/ml. The nicotine concentrations present in our stock (10 puffs/100 ml) extract solutions were determined to be the following: E0, below limit of detection; N12 = 0.254±0.026; E12 = 0.535±0.021; E18 = 1.715± 0.009; N18 = 1.957±0.030; MS = 15.419±0.134 μg/ml. The average nicotine yield for one cigarette was 1.542 mg, which is within the range described for reference 3R4F cigarettes smoked under International Standards Organization (0.707 to 1.84 mg) or HCI (1.90 mg) smoking conditions [27, 42, 43].

### Cell culture

All cell lines were cultured under standard conditions [44]. Human epithelial normal bronchial cells (Nuli1) were cultured in serum free Airway Epithelial Cell Basal Medium (ATCC CRL-4011), with Bronchial Epithelial Cell Growth Kit additives (ATCC PCS-300-040). The human premalignant dysplastic oral mucosal keratinocyte cells (POE9n) were cultured in keratinocyte serum free medium supplemented with 25 μg of bovine pituitary extract (BPE) per ml, 0.2 ng of EGF per ml, and 0.4 mM CaCl_2_ [45, 46]. Human oral squamous cell carcinoma (UM-SCC-1) cells were cultured in high glucose Dulbecco’s modified Eagle’s medium (DMEM) supplemented with 10% fetal bovine serum [47]. Cell line verification was performed by short-tandem repeat-based DNA profiling (CellCheck Cell line authentication, IDEXX Bioresearch, Columbia, MO, USA).

### E-cigarette aerosol and tobacco smoke exposure

For short-term experiments, epithelial cancer and non-cancer cells were exposed for 1 h to diverse doses of EC aerosol extract (equivalent to 1, 10 or 100 puffs/5 L). The EC doses used include doses representative of the approximate number of puffs reported by EC users: 13 puffs/vaping session [40] or 120–180/day [7, 48] and are indicated as puffs per 5 L (the typical blood volume of an adult). To mimic chronic genotoxic exposure [49–51], cells were treated every other day for 2 weeks with 10 puffs/5 L of EC aerosol extract. Control cells were exposed to vehicle only. Mainstream smoke extract was used for comparison at a dose equivalent to 10 puffs/5 L (~ 1 cigarette), which we have previously shown to cause significant DNA damage [52].

### Quantification of DNA damage

Total genomic DNA was isolated according to Mullenbach *et al*. [53], and following previously described steps to reduce artifactual DNA damage [54]. DNA damage was quantified using two distinct methods: a PCR based assay (q-PADDA) which detects many types of DNA damage with high sensitivity [52, 54], and a colorimetric based assay (HT 8-oxo-dG ELISA Kit II, Trevigen, MD) which detects exclusively 8-hydroxy-2’-deoxyguanosine (8-oxo-dG) lesions. 8-oxo-dG is one of the major products of DNA oxidation. The primer-anchored DNA damage detection assay (q-PADDA) was performed as we previously described [52]. We chose to quantify DNA damage within the transcribed strand (TS) and non-transcribed strand (NTS) of *TP53* (commonly referred as *p53*), because *p53* is the most frequently mutated gene in human cancer [55] and is mutated in nearly all smoking related cancers.[56, 57] For DNA damage quantification, as well as for all other analysis described below, we performed three independent experiments, each with 3–6 technical replicates.

### ROS, TAC and MTT assays

The activity of hydroxyl, peroxyl and other reactive oxygen species (ROS) within the cell was determined using a standard 2’,7’–dichlorofluorescin diacetate (DCFDA) assay (Abcam, MA). The total cellular antioxidant activity was measured using a standard kit (Cayman Chemical Company, MI), which relies on the ability of antioxidants in the sample to inhibit the oxidation of ABTS (2,2'-azino-di-[3-ethylbenzthiazoline sulphonate]). Cell viability was measured by MTT (3-[4,5-dimethylthiazol-2-yl]-2,5-diphenyltetrazolium bromide) assay (Invitrogen, CA) as we previously described [52]. All assays were performed as recommended by the respective manufacturer. Three independent experiments were performed, each with at least three technical replicates.

### Real-time RT-PCR analysis

Total RNA was isolated from cells using TRIzol reagent (Invitrogen) and subjected to reverse transcription with Superscript^TM^ II RNase H—Reverse Transcriptase and random hexanucleotide primers (Invitrogen). The cDNA was subsequently used for real-time RT-PCR using gene specific primers (ERCC1-512F, GGCGACGTAATTCCCGACT; ERCC1-596R, TAGCGGAGGCTGAGGAACA; OGG1-714F, AAATTCCAAGGTGTGCGACTG; OGG1-796R, GCGATGTTGTTGTTGGAGGA). β-actin expression was used as a normalization control as we previously described [58]. The changes in mRNA were expressed as fold change relative to untreated cells.

### Western blot analysis

Protein was extracted in radioimmuno precipitation assay buffer (RIPA), sonicated, and centrifuged at 12,000 r.p.m. for 10 min at 4^°^C. Total protein (40 μg) from each sample was fractionated on 10% sodium dodecyl sulfate-polyacrylamide gel electrophoresis (SDS-PAGE) and electrotransferred on to a PVDF (polyvinylene difluride) membrane. The membranes were blocked for 1 h with 5% non-fat milk and incubated overnight at 4^°^C with primary antibodies [ERCC1 (sc-56673), OGG1 (sc-376935) or actin (sc-1616); Santa Cruz Biotechnology, CA]. The membranes were then washed thrice in Tris-Buffered Saline and Tween 20 (TBS-T). This was followed by incubation with secondary antibodies coupled with HRP (Santa Cruz Biotechnology) for 1 h at room temperature, and thrice washed in TBS-T. Immunoreactive antibody–antigen complexes were visualized with the enhanced chemiluminescence reagents (Pierce Biotechnology, IL). The signals were detected using the ChemiDoc^TM^ touch imaging system (BioRad, CA) and quantified using Image lab software (BioRad, CA). Protein expression was normalized using actin as control.

### Statistical analysis

Data were compiled in Excel (Microsoft) files and statistical analyses were performed using SAS/STAT Version 9.1 (SAS Institute Inc.). Independent means were compared using unpaired Student's *t* tests whose degrees of freedom were corrected, when appropriate, for inequality of variance. We considered *p* < 0.05 to be statistically significant.

## Results

### EC aerosols induce a dose-dependent increase in DNA damage

**Fig 1:**
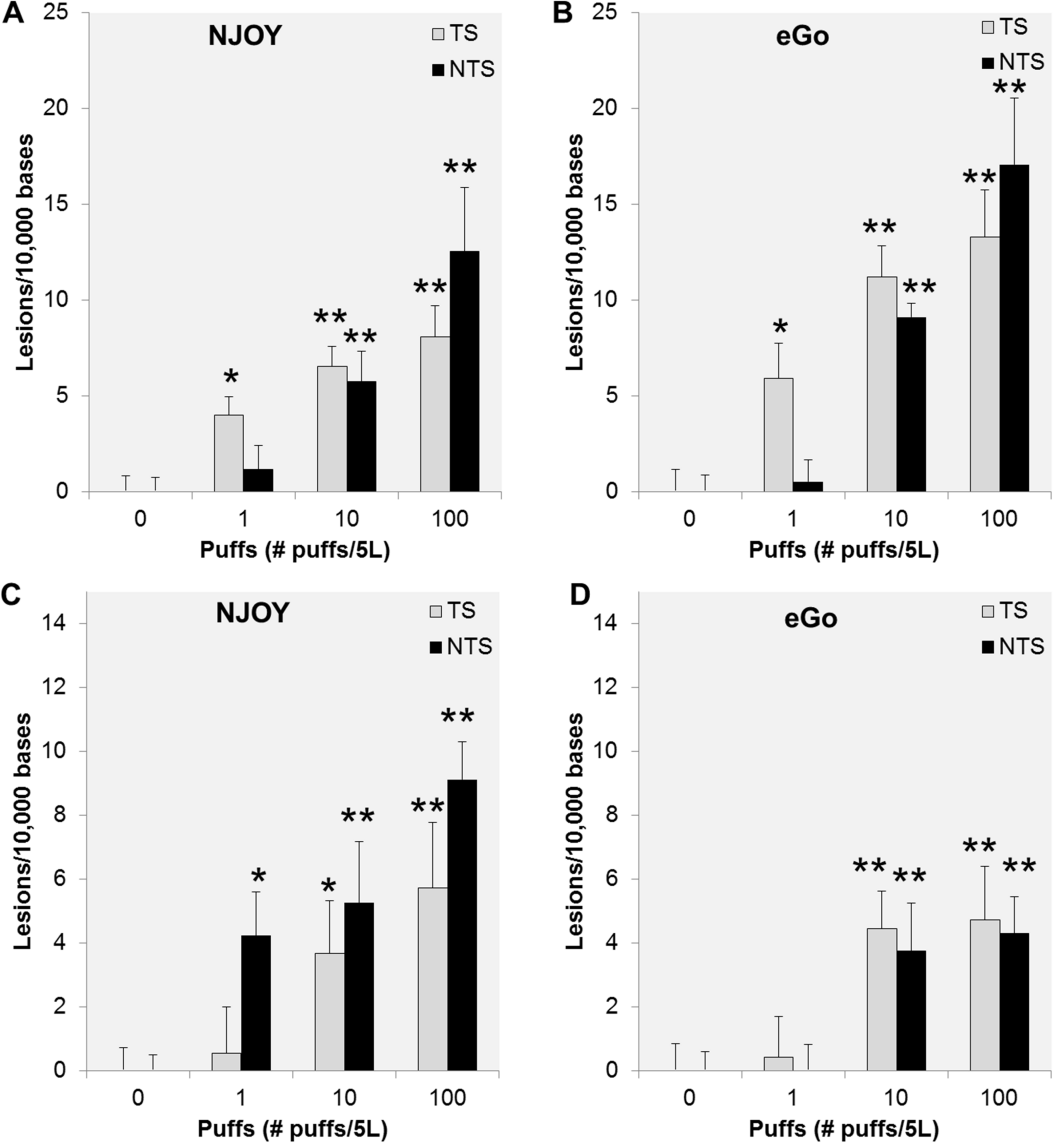
Dose-dependent increase in DNA damage in cells exposed to EC aerosol extracts. UM-SCC-1 (A and B) and NuLi1 (C and D) cells were exposed for 1 h to increasing doses of NJOY (N18) or eGo (E18) and DNA damage quantified by q-PADDA within the transcribed (TS) and non-transcribed (NTS) strands of the *TP53* gene. Data are represented as mean ± SEM. *p<0.05, **p<0.01.

EC aerosols contain several potential toxicants and have been reported to share various adverse effects with tobacco smoke including: causing oxidative stress [32, 33] and eliciting bronchial patterns of gene expression similar to tobacco smoke [59, 60]. Therefore, it is essential to determine whether exposure to EC aerosol can be a significant source of DNA damage to oral and lung epithelial cells. We have previously reported that exposure of human oral epithelial cancer cells (UM-SCC-1) to very low doses of mainstream smoke extract causes significant DNA damage measurable by q-PADDA [52]. Our initial aim was to assess whether EC aerosol has a similar effect on oral and lung epithelial cells. Hence, we first exposed UM-SCC-1 cells for 1 h to increasing doses of aerosol extracts obtained from two distinct brands of ECs and measured DNA damage using q-PADDA. This assay has high sensitivity to detect many types of DNA damage including alkylative and oxidative lesions [52, 54], potentially caused by formaldehyde and reactive oxygen species present in EC aerosol. Both EC aerosol extracts induced significant DNA damage (*p*<0.001) in UM-SCC-1 cells when compared to the unexposed control cells (Fig 1A and 1B). A significant increase in DNA damage was also observed in NuLi1 cells, an immortalized cell line established from normal bronchus epithelium, after 1 h exposure to either EC aerosol extract (Fig 1C and 1D). Remarkably, the increase in DNA damage (*p*<0.001) was consistently observed in both *p53* DNA strands and in both cell lines for doses equivalent to 10 or more EC puffs/5L (Fig 1). Moreover, a dose-dependent increase in DNA damage was also observed in both cell lines and for both EC extracts (Fig 1). No cell death was observed for either of the doses used (S1 Fig). By comparison with our previously published data [52], the levels of DNA damage induced by EC extracts are lower than those induced by mainstream smoke extracts. Altogether, our data show that EC aerosol can cause significant DNA damage in human oral and lung epithelial cells.

### DNA damage induced by EC aerosols is nicotine independent

**Fig 2:**
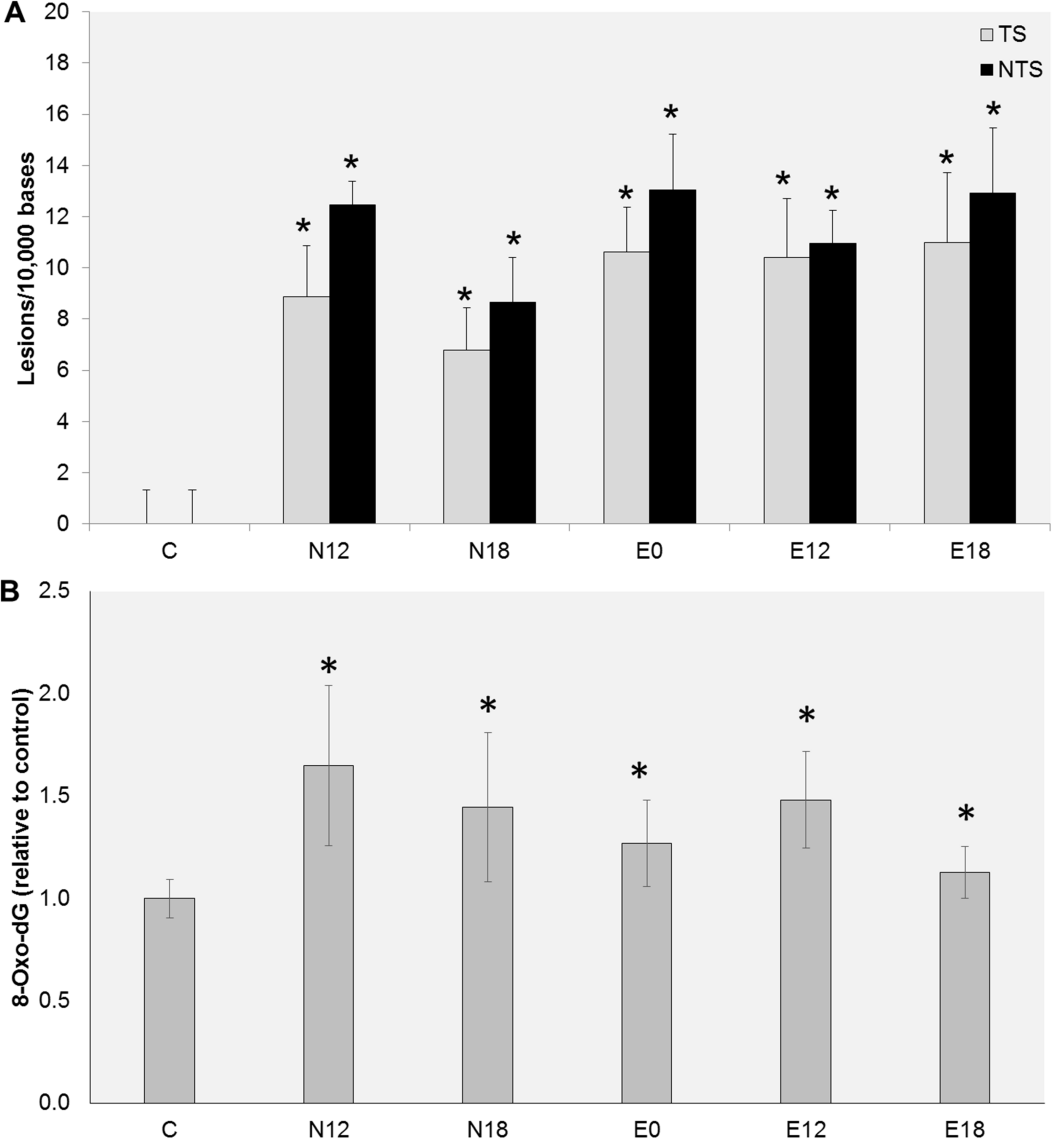
Effect of nicotine on DNA damage levels. UM-SCC-1 cells were exposed for 1 h to EC aerosol extracts obtained from e-liquids with different nicotine concentrations and DNA damage quantified by q-PADDA (A) or ELISA (B). Data are represented as mean ± SEM. *p<0.05.

To determine whether the observed DNA damage is dependent of the level of nicotine present in the EC aerosol, cells were exposed to extracts obtained from e-liquids containing diverse nicotine concentrations (0, 12 or 18 mg/ml). All extracts induced significant levels of DNA damage within *p53* (Fig 2A). Yet, EC aerosols obtained from e-liquids lacking nicotine caused levels of DNA damage similar to those obtained from e-liquids containing 12 or 18 mg/ml of nicotine (Fig 2A). EC aerosol have been reported to contain high levels of reactive oxygen species [32, 33]. Therefore, we determined whether exposure to EC aerosol extracts causes oxidative damage. For that purpose we chose to quantify the levels of 8-oxo-dG, one of the most frequent and most mutagenic oxidative DNA lesions. As shown in Fig 2B, all EC extracts induced significant levels of 8-oxo-dG. Consistent with the data observed with q-PADDA, the levels of damage induced by the diverse extracts were similar and independent of the concentration of nicotine present in the e-liquid from which the aerosols were derived (Fig 2B). Altogether, these data suggest that other components, rather than nicotine, are responsible for the DNA damage induced by EC aerosol extracts.

### Chronic exposure to EC aerosols causes significant oxidative DNA damage

**Fig 3:**
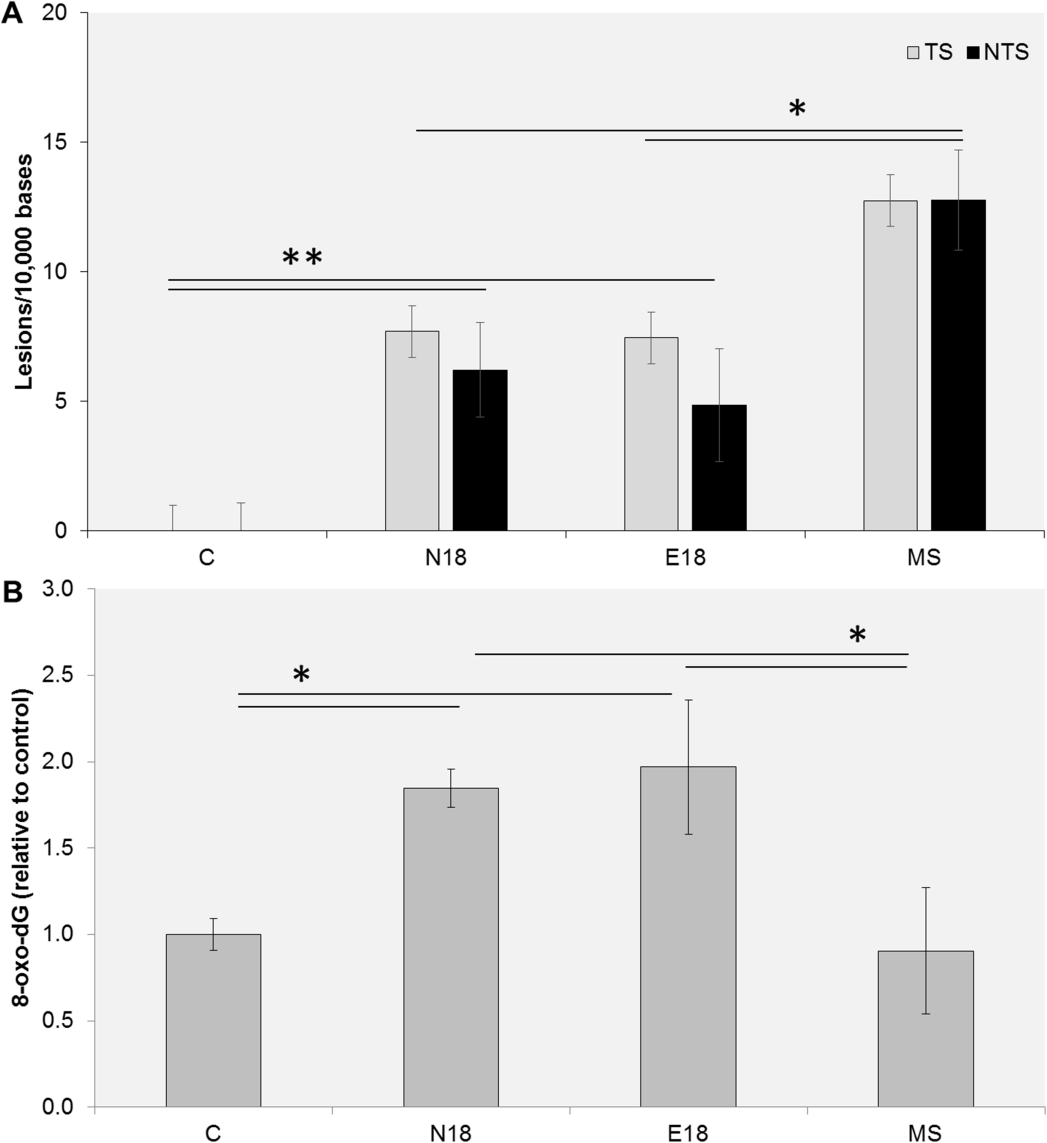
DNA damage levels measured after long-term exposure to EC aerosol or MS smoke extracts. UM-SCC-1 cells were exposed for 2 weeks to EC aerosol (N18 and E18) or MS smoke extracts and DNA damage quantified by q-PADDA (A) or ELISA (B). Data are represented as mean ± SEM. *p<0.05; **p<0.01.

DNA damage is the main initiator of cancer [61] and plays a key role in the pathogenesis of aging, neurodegenerative, pulmonary, and cardiovascular diseases [62, 63]. To compare the potential DNA damaging effects of long-term exposure to EC aerosols and tobacco smoke extracts, we measured overall DNA damage within *p53* and quantified total 8-oxo-dG DNA lesions in cells exposed for 2 weeks to either EC aerosol (N18 or E18) or mainstream (MS) smoke extracts. Significant levels of DNA damage within *p53* were observed in cells exposed to EC extracts relative to unexposed control cells (Fig 3A). Consistent with the data observed after acute exposure, we also observed significantly lower levels of DNA damage within *p53* in cells exposed to EC aerosol extracts than in cells exposed to mainstream smoke extracts. Cells exposed to EC aerosol extract also had significantly higher levels of 8-oxo-dG, than unexposed cells (Fig 3B). These data show that chronic exposure to EC aerosol can induce significant DNA damage, including highly mutagenic oxidative damage. Intriguingly, the observed levels of 8-oxo-dG in UM-SCC-1 cells were higher after long-term exposure to EC aerosol than to mainstream smoke extract (Fig 3B). These data suggest that either EC aerosols cause more oxidative stress than mainstream tobacco smoke, or cells exposed to EC fail to activate cellular pathways that prevent oxidative DNA damage.

### EC aerosols induce significant ROS and decrease the cellular total antioxidant capacity

**Fig 4:**
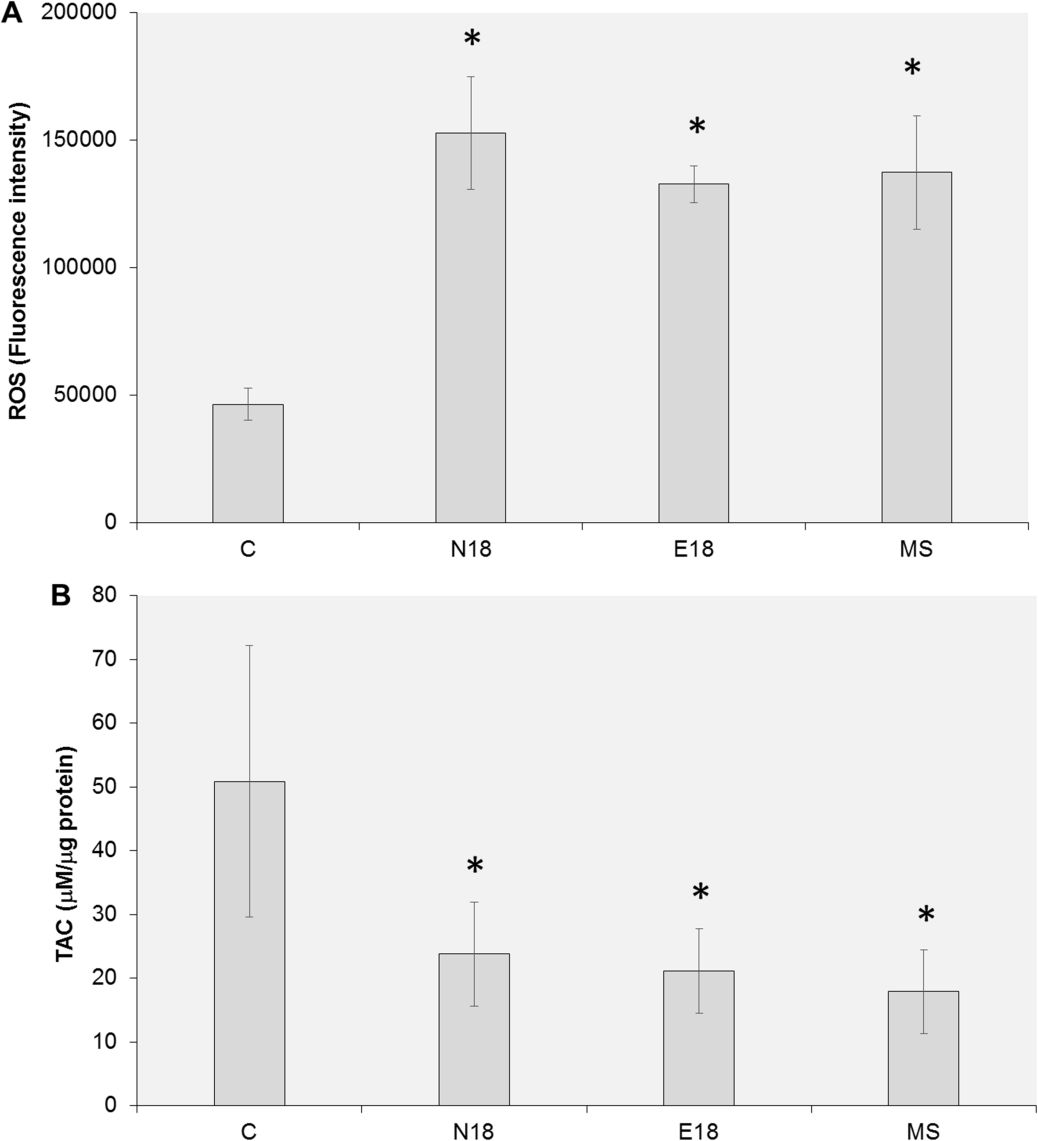
Exposure to EC aerosol increases cellular ROS and decreases TAC. UM-SCC-1 cells were exposed for 2 weeks to EC aerosol (N18 and E18) or MS smoke extracts and the cellular levels of ROS (A) and TAC (B) were measured using standard methods. Data are represented as mean ± SEM. *p<0.05.

To investigate the mechanisms contributing to our observations, we quantified the intracellular levels of ROS in UM-SCC-1 cells exposed to EC aerosol or mainstream smoke extracts for 2 weeks. We observed a significant increase in the intracellular levels of ROS after exposure to EC extracts (Fig 4A). Similar levels of ROS were also observed after long-term exposure to mainstream smoke (Fig 4A). Then, we measured the cellular total antioxidant capacity after long-term exposure to EC aerosol or mainstream smoke extracts. Consistent with the observed increase in cellular ROS, we observed a significant decrease in the total antioxidant capacity of cells exposed to EC aerosol (Fig 4B). There was no significant difference in TAC levels between EC exposed cells and MS exposed cells (Fig 4B). These data suggest that at the doses tested, EC and mainstream smoke induce similar levels of oxidative stress.

### EC aerosols decrease the levels of proteins essential for the removal of DNA damage

**Fig 5:**
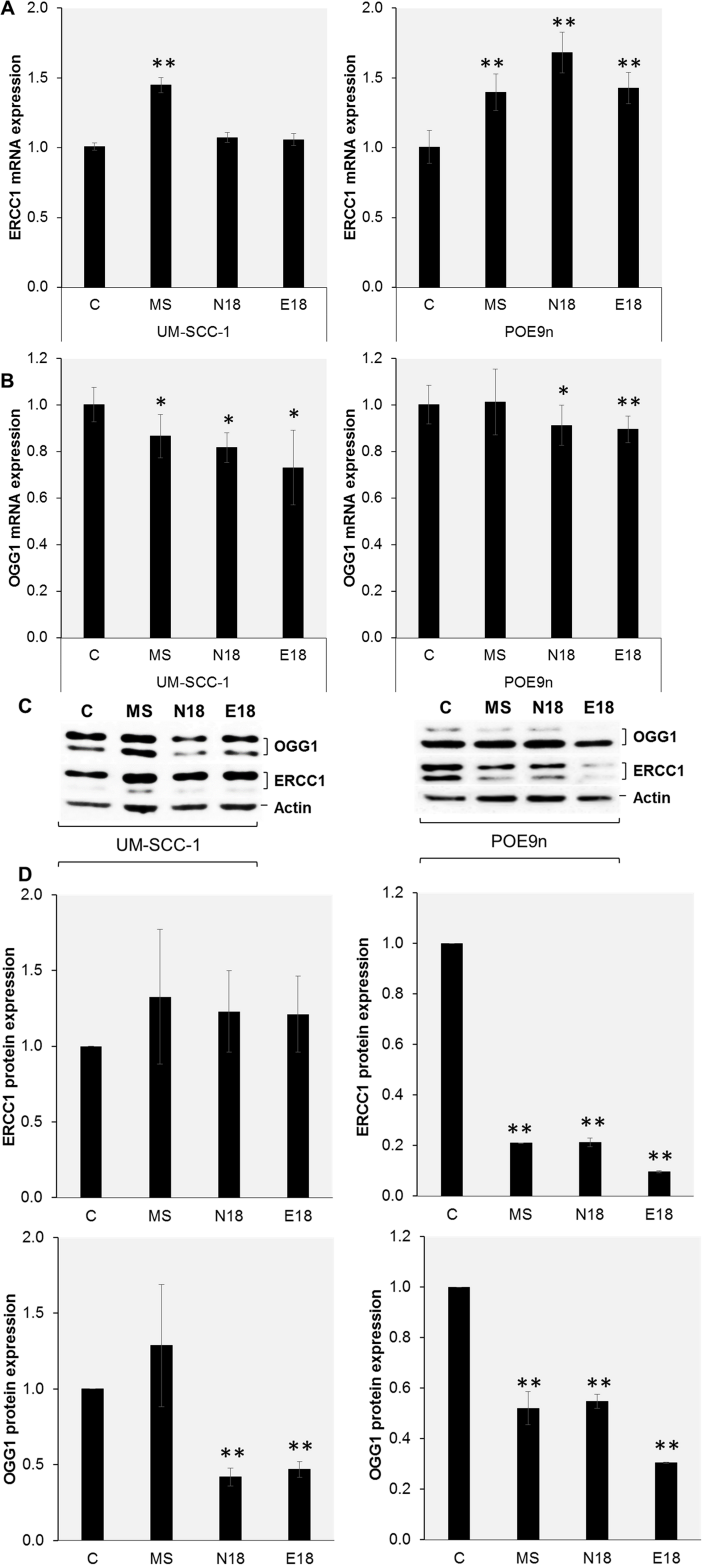
Exposure to EC aerosol changes the expression of DNA repair proteins. UM-SCC-1 and POE9n cells were exposed for 2 weeks to EC aerosol or MS smoke extracts and the levels of mRNA (A,B) and protein (C) were measured by real-time RT-PCR and western blot, respectively. Protein expression after chronic exposure to EC aerosol or MS smoke extracts was quantified and compared to non-exposed cells (D). Data are represented as mean ± SD. *p<0.05; **p<0.01.

Aldehydes and free radicals, such as those reported in EC aerosols and tobacco smoke, are known to cause diverse types of DNA damage which are repaired mainly by nucleotide excision repair (NER) and base excision repair (BER). Hence, we assessed whether chronic exposure to EC aerosol and mainstream smoke extracts modifies the expression of ERCC1 (excision repair cross-complementation group 1), a NER protein essential for the removal of bulky DNA damage, and OGG1 (8-oxoguanine-DNA glycosylase), a BER protein essential for the removal of oxidative DNA lesions. Using real-time RT-PCR analysis, we observed that *ERCC1* mRNA expression was significantly increased in cells after exposure to MS smoke, compared to unexposed control cells (Fig 5A). A similar trend was observed after exposure to EC extracts, but the data only reached significance in POE9n cells (Fig 5A). In contrast, we observed that exposure to EC aerosol (N18 and E18) caused a significant decrease (p<0.01) in the expression of *OGG1* mRNA in both cell lines, compared to unexposed control cells (Fig 5B). Exposure to MS smoke lead to a significant decrease in *OGG1* mRNA only in UM-SCC-1 cells (Fig 5B). To assess whether exposure to EC aerosol and MS smoke extracts had an impact on protein availability for the removal of the diverse types of DNA damage, we performed western blot analysis. We observed that chronic exposure to EC aerosol or mainstream smoke extracts caused a significant reduction in ERCC1 protein in POE9n cells, but did not significantly change the levels of ERCC1 protein expression in UM-SCC-1 cells (Fig 5C). Most importantly, consistent with our mRNA analysis, chronic exposure of either cell line to EC aerosol extracts led to a significant decrease in the expression of the OGG1 protein (Fig 5C and 5D). Chronic exposure of POE9n to MS smoke extracts also lead to a significant decrease in the expression of OGG1 protein (Fig 5C and 5D). No significant changes in OGG1 protein levels were observed after exposure of UM-SCC-1 cells to MS smoke extracts (Fig 5C and 5D). These data suggest that the significant decrease in the expression of OGG1 protein might lead to a significant decrease in the repair of 8-oxo-dG, and contribute to the observed high levels of 8-oxo-dG lesions observed after chronic exposure to EC aerosol extracts. Overall, these data show that chronic exposure to EC aerosols can lead to a significant reduction in the level of OGG1 and ERCC1, and consequently might reduce the overall repair capacity of the two main pathways responsible for the repair of oxidative and bulky DNA damage: basic excision and nucleotide excision repair.

## Discussion

Active and passive smoking constitute a significant public health problem, as tobacco smoke is the leading preventable cause of morbidity and mortality [64]. ECs have been promoted as a smoking cessation aid and, based on limited data, are perceived as less harmful to one’s health than traditional cigarettes. However, the study of the potential long-term health effects of EC use is lacking. In this study, we examined the effects of exposure to EC aerosol extracts on DNA damage in human oral and lung epithelial cells. Moreover, we investigated the effects of long-term exposure to EC aerosol extracts on the main cellular mechanisms that modulate the levels of oxidative stress and DNA damage: total antioxidant capacity, cellular ROS and DNA damage repair. Parallel studies with mainstream smoke allowed for side-by-side comparison. Of major public health relevance, all the EC aerosols used in this study induced significant DNA damage, including high levels of 8-oxo-dG, a highly mutagenic DNA lesion.

The puffing regimen used for EC aerosol and MS smoke extracts is similar to the puffing regimen that has been recommended for EC aerosol studies [38]. The smoking regimen used to obtain MS smoke extract (two 50 ml puffs/minute) mimics the reported human puffing profiles for cigarettes with more than 14 mg tar [37] and is similar to the Health Canada Intensive smoking standard conditions (two 55±5 ml puffs per minute). These conditions have been reported to generate higher amount of smoke per cigarette than the ISO smoking conditions (one 35 ml puff per minute), however the amount of toxicants and the *in vitro* toxicity per mg of nicotine have been reported to be generally lower under HCI than under ISO smoking conditions [43].

The aerosol doses used in our study (1 to 100 puffs/5 L) encompass those obtained by experienced EC users during a vaping session. For example, it has been reported that eGo EC users vape on average 13 puffs and consume 62 ± 16 mg e-liquid in 5 min (similar to the time needed to smoke one tobacco cigarette) vaping session [40]. For chronic exposure, we chose to use EC aerosol extracts at a dose equivalent to 10 puffs/5L. Based on the nicotine measured in our extracts, which varied with the EC device and the e-liquid nicotine concentration, 10 puffs of EC aerosol extract per 5L lead to approximately zero (E0), 5.1 (N12), 10.7 (E12), 34.3 (E18), or 39.1 (N18) ng/ml of nicotine. These doses are representative of the nicotine concentrations we observed in the plasma of EC users immediately after a 10 puff session (4.5–30.4 ng/ml) or a two hour *ad libitum* vaping session (10.7–36.4 ng/ml) [65]. Moreover, they encompass the nicotine levels reported in the plasma of smokers and are significantly lower than the average dose present in the oral cavity of vapers and smokers, which due to nicotine concentration in saliva can be up to 100-fold higher than in the plasma [66].

To the best of our knowledge, our study is the first to show that short-term exposure to increasing doses of EC aerosol induces a dose-dependent increase in DNA damage. Moreover, we have shown that long-term exposure to doses of EC extract, similar to those experienced by EC users [65], leads to significant levels of DNA damage. Previously, a single study has reported that exposure to EC aerosol extracts caused extensive DNA strand-breaks [67]. However, the biological relevance of this study is hampered by the fact that the authors used an extremely high dose of EC aerosol extracts (1% by volume), reported by the authors to be equivalent to 500 μM of nicotine and to cause very high cell death (49% to 66% in non-cancer cells). Cell death leads to DNA breakage and is a major confounding variable when DNA damage is measured exclusively in function of detectable strand breaks, as it was reported by Yu et al. (2016). Using doses up to 100 puffs/5L, which is well above the average puffing at a single vaping session, we observed no cell death in cancer and non-cancer cells. Our data is consistent with all other toxicology studies using EC aerosols, which have shown that, in contrast with EC e-liquids [68–71], EC aerosols cause little or no cell death [39, 68, 72].

In our study, we used two completely distinct approaches to measure DNA damage: a very sensitive PCR based assay (q-PADDA) that quantifies a broad spectra of DNA lesions (including oxidative, alkylative and bulky lesions) [52, 54], and an ELISA assay that detects a specific type of oxidative DNA damage (8-oxo-dG). DNA damage precedes mutation and varies significantly by genomic area. Therefore, for q-PADDA assay, we chose to quantify damage in *p53*, because *p53* is the most frequently mutated gene in human cancer [55], and is mutated in nearly all smoking related cancers [56]. Our data consistently showed that both short-term and long-term exposure to EC aerosol extracts led to a significant increase in DNA damage which is detectable by both methods, ELISA and q-PADDA.

There have been no previous studies comparing side-by-side the effects of similar doses of EC aerosol and mainstream smoke extracts on overall DNA damage. Nonetheless, a single study has reported that tobacco smoke causes significantly higher levels of double-strand breaks (a specific type of DNA damage) than EC aerosol [73]. Tobacco smoke is a complex mixture of more than 7000 chemicals: hundreds of these are hazardous, and at least 69 are known to damage DNA and cause cancer [74]. EC aerosols have been reported to contain potentially toxic compounds, but overall the number of genotoxic substances and their concentration, for the most part, has been reported to be significantly lower than those in cigarette smoke [20, 24–26]. In agreement with these data, we showed that both short-term and long-term exposure to EC extract led to overall less DNA damage than the equivalent exposure to mainstream smoke.

A single cigarette puff of tobacco smoke contains over a 10^14^ free radicals [75], and causes a 35–50% increase in oxidative DNA damage [74]. EC aerosols have also been reported to contain high levels of free radicals [32–34] and shown to cause oxidative stress and inflammatory responses [32, 33, 39, 76]. Yet, no previous studies have reported whether or not EC aerosols cause oxidative DNA damage. Here, we showed that both short-term and long-term exposure to EC aerosol extracts induced a significant increase in the levels of 8-oxo-dG, one of the major DNA lesions formed from reactive oxygen species (ROS). These data are of high concern, as 8-oxo-dG base modification is highly mutagenic and if not recognized and repaired it may cause GC to TA transversions [77, 78]. Moreover, oxidative DNA lesions have been proposed to cause the vast majority of human mutations [79]. Our data is in apparent contradiction with the fact that ECs have been deemed non-mutagenic to Ames Salmonella tester strains TA98 and TA100 [80, 81]. However, none of the previous studies included the strains TA102 or TA104 which have the highest sensitivity for the detection of mutagenesis induced by oxidative or cross-linking agents [82]. TA104 has also been reported to be particularly sensitive to carbonyl based compounds [83], as those reported to be present in EC aerosol. Moreover, the Organization for Economic Co-operation and Development (OECD) recommends testing a battery of at least five tester strains to capture the full range of chemical interactions and mutagenic events acting via different modes of action[81]. Another variable to consider, is the fact that toxicant yield varies with EC brand, product model, e-liquid flavor, e-liquid solvent, and device power [19, 21, 23, 84, 85] so it is not possible to directly compare our study to others using different ECs or e-liquids. Nonetheless, altogether these data stress the need to further investigate whether the oxidative damage induced by EC aerosol increases mutagenicity.

Oxidative DNA damage reflects not only exposure to specific genotoxics, but also the cellular capacity for antioxidant detoxification and DNA repair. Tobacco smoke has been previously shown to reduce the antioxidant capacity of tissues [86]. Here, we showed that like MS smoke, long-term exposure to EC aerosol significantly increases cellular ROS and decreases total cellular antioxidant capacity. These observations are very important as they pinpoint additional mechanisms, besides the genotoxic content of EC aerosols, by which exposure to EC aerosol might contribute to increase DNA damage.

To deal with the large variety of DNA lesions induced by endogenous and exogenous genotoxics, human cells have developed a network of DNA repair mechanisms, involving more than 150 genes. Among the major types of DNA repair mechanisms, nucleotide excision repair (NER) is the most versatile repair pathway in the cell. NER is the primary mechanism for the removal of bulky DNA adducts that significantly distort the DNA helix structure, such as those induced by tobacco smoke [87, 88]. NER also plays an important role in the repair of oxidative damage. Basic excision repair (BER) is the main pathway for the removal of small base lesions caused by reactive oxygen and nitrogen species, as well as by alkylating agents [87], such as those reported in EC aerosol and tobacco smoke [15, 22]. Tobacco smoke has previously been shown to reduce the rate of DNA damage repair [89]. Here, we show for the first time that exposure to EC aerosol extracts can also lead to a significant decrease in the expression of ERCC1 and OGG1, two DNA excision repair proteins that are rate limiting for the efficiency of NER and BER, respectively. ERCC1 forms a complex with XPF protein forming a nuclease that is essential for incision of a DNA lesion during nucleotide excision repair. The ERCC1-XPF nuclease also participates in the repair of DNA double-strand breaks and crosslinks. NER deficiency results in the skin cancer-prone inherited disease xeroderma pigmentosum (XP) [87]. Polymorphic variations on *ERCC1* gene and low levels of the ERCC1 enzyme have also been shown to be a marker for susceptibility to tobacco-related cancers [90]. OGG1 is the main enzyme responsible for the removal of 8-oxo-dG. Alteration in the levels of OGG1 and polymorphisms in the *OGG1* gene modulate BER capacity and are known risk factors for human cancer [91]. The fact that EC aerosols used in this study, like MS smoke, can lead to a significant decrease in the expression of ERCC1 and OGG1, suggest yet another mechanism by which exposure to EC aerosol can contribute to DNA damage, and therefore increase cancer risk.

These observations have significant implications not only for the repair of DNA damage induced by exposure to EC aerosol, but also for the repair of DNA damage induced by other toxicants that EC users and bystanders might be exposed to.

Due to the high number of variables studied and length of exposure, our study used established cell lines instead of primary cells or tissues. Established cell lines might lack metabolic competency which can affect their ability to bioactivate and/or detoxify xenobiotics. All cell lines used in this study have an intact wild-type *p53* gene [92], but due to the presence of HPV E6/E7 or a splice mutation (UM-SCC-1) express low levels of wild-type p53 protein [93] and might have impaired DNA repair capacity. Cell lines with defective DNA repair are anticipated to have higher levels of basal DNA damage than those with effective DNA repair, making it harder to detect small increases in DNA damage. Yet, our data clearly shows that under the conditions tested exposure to EC aerosol induces significant DNA damage. Whether these observations reflect mainly an *in vitro* exposure or is a common event occurring in EC users is currently being investigated in our laboratory.

In summary, here we report for the first time that exposure to EC aerosol extracts can cause significant levels of DNA damage, including high levels of 8-oxo-dG, an oxidative and highly mutagenic DNA damage lesion. Even so, the overall EC-induced DNA damage levels were in general lower than the levels of damage induced by mainstream smoke extracts. By examining the mechanisms that modulate DNA damage, we identified an increase in cellular ROS, a decrease in TAC, and a decrease in the expression of proteins essential for DNA damage repair as novel mechanisms by which exposure to EC aerosol can cause DNA damage and potentially increase cancer risk. Our study emphasizes the further need to investigate the health consequences of exposure to EC aerosols and highlights the extreme importance in regulating ECs and the exposure to EC aerosol.

## Supporting information

S1 Fig Exposure to EC aerosol extracts does not cause cell death. UM-SCC-1 and NuLi1 cells were continuously exposed to 10 or 100 puffs/5L of EC aerosol extracts and cell viability was determined by MTT assay at 96 h. Data are represented as mean ± SD.
(TIF)

S1 File Analytical determination of nicotine content. (PDF)
