# Supplementary figures and images for: Electronic cigarette aerosols suppress cellular antioxidant defenses and induce significant oxidative DNA damage
Source: PLoS One. 2017 May 18;12(5):e0177780. doi: 10.1371/journal.pone.0177780 (PMC5436899; doi:10.1371/journal.pone.0177780)

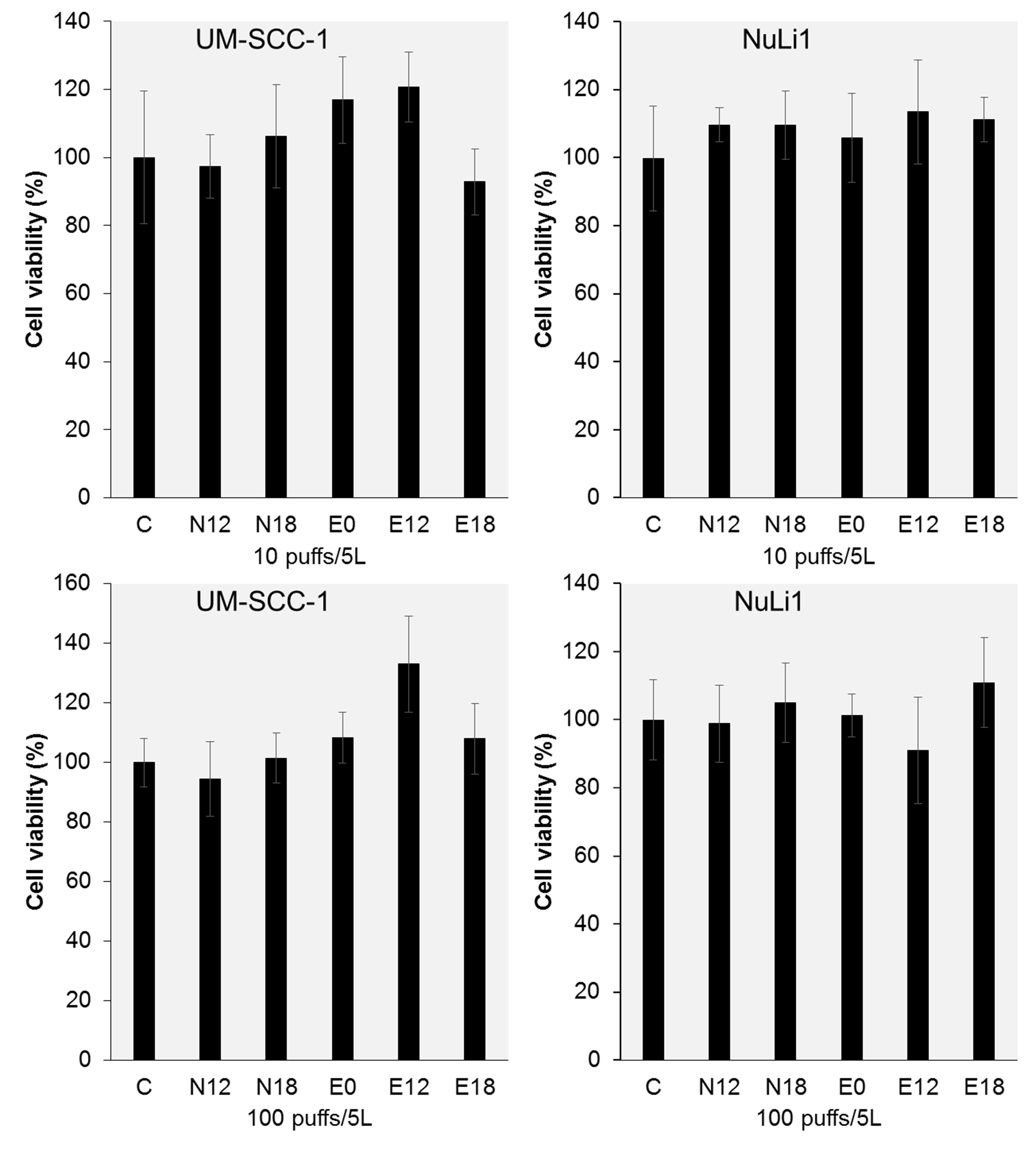

Supplement: S1 Fig — UM-SCC-1 and NuLi1 cells were continuously exposed to 10 or 100 puffs/5L of EC aerosol extracts and cell viability was determined by MTT assay at 96 h. Data are represented as mean ± SD. (TIF) [file pone.0177780.s001.tif]
