# Supplementary material for: Electronic cigarette aerosols suppress cellular antioxidant defenses and induce significant oxidative DNA damage
Source: PLoS One. 2017 May 18;12(5):e0177780. doi: 10.1371/journal.pone.0177780 (PMC5436899; doi:10.1371/journal.pone.0177780)
Supplement: S1 File — (PDF) [file pone.0177780.s002.pdf]

Analytical Determination of Nicotine Content:

The determination of nicotine content was performed by Gas Chromatography Mass Spectroscopy (GCMS) analysis using a pH assisted dichloromethane (DCM) solvent exchange technique described by Gellner et al [1] but modified for smaller volume analysis more suitable for typical molecular biological laboratories. Before extraction prepare DCM solution with 50 µg/mL internal standard (ISTD, quinoline). In a large microfuge tube, 500 µL aerosol extract in HEPES buffer was treated with 10M 50 µL sodium hydroxide (NaOH) to bring pH above the pKa of nicotine (pH greater than 8). This reduces water solubility and enhances partitioning to organic extraction solvent DCM. Add 1.0 mL DCM and vortex 60 s. Allow water and organic phases to separate completely then discard water phase (top). Add 500 µL 1M hydrochloric acid (HCl) to vial and votex 60 s. The aqueous extraction at pH < 3 is highly favored due to the double protonation of Nicotine at each nitrogen center. Discard the DCM layer (bottom). Add 100 µL 10M NaOH to neutralize the HCl and bring pH >8, vortex 10 s. Add 500 µL DCM+ISTD and vortex 60 s. Transfer DCM layer (bottom) to glass vial with PTFE lined lid and store in freezer until analysis. Solvent evaporation will occur even at low temperature and samples should be analyzed as soon as reasonably possible, for this study within 48 hrs of extraction. Addition of ISTD to the final extraction step facilitates correction for solvent evaporation and injection variability during analysis.

Gas Chromatography Mass Spectroscopy (GCMS) analysis was conducted using an Agilent 6890 GC with 5973 quadrapole Mass Selective Detector using the following parameters. Inlet: 280C, splitless, constant pressure. Column: Restek Rtx-5 30 m, 0.32 mm inside diameter, 0.5 µm film thickness, 1.5 mL/min flow rate. Oven: Initial 50 C, ramp 25 C/min to 170 C, ramp 40 C/min to 270 C, hold 2 min, MSD: scan 14-220 m/z. Quantitation was performed using the 84 m/z ion with 133 and 162 m/z qualifying ions. Due to the selective nature of the extraction procedure, clear separation and sharp peak efficiency was observed for nicotine (RT=5.6 min) and ISTD (RT=5.1 min) in all samples, including main stream smoke. To calibrate the GCMS, nicotine in HEPES solutions were prepared from 50 - 0.098 µg/mL following a 2-fold serial dilution. These solutions were extracted and analyzed using the procedure and instrument parameters described above. Separate solutions of nicotine in HEPES were prepared, extracted and analyzed to verify the accuracy of the calibration. The method detection limit (MDL) of 0.076 µg/mL was estimated from 11 replicate analyses of nicotine in HEPES at 0.39 µg/mL using the EPA approach[2]. Lower limit of quantification (LOQ) was set a 3MDL = 0.227 µg/mL. Blank verification was performed on 10 samples of HEPES solution used to prepare the nicotine calibration solutions and tissue culture samples. Blanks were below MDL.

1. Gellner, C. A.; Reynaga, D. D.; Leslie, F. M., Cigarette Smoke Extract: A Preclinical Model of Tobacco Dependence. *Current Protocols in Neuroscience* **2016**, 9.54. 1-9.54. 10.

2. Definition and Procedure for the Determination of the Method Detection Limit, 40 C.F.R. § 136 App. B (2016)
